# Two bHLH Transcription Factor Genes *AhWSC1a* and *AhWSC1b* Act as Gatekeepers of Testa Pigmentation, Preventing White Seed Coats in Peanuts

**DOI:** 10.3390/plants15020304

**Published:** 2026-01-20

**Authors:** Guanghui Chen, Yan Ren, Lin Liu, Ping Xu, Yueyi Tang, Hui Wang, Heng Wang, Jiaxin Tan, Lijun Wu, Shuangling Li, Tianying Yu, Zhiwei Wang, Jiancheng Zhang, Mei Yuan

**Affiliations:** 1 Shandong Peanut Research Institute, Shandong Academy of Agricultural Sciences, Qingdao 266100, China; ghchen@sdpeanut.ac.cn (G.C.); renyan@sdpeanut.ac.cn (Y.R.); yueyit@126.com (Y.T.); tanjiaxin0246@163.com (J.T.); wljd126@126.com (L.W.); lisl7105@163.com (S.L.); wzw_1983@163.com (Z.W.); 2 College of Life Science, Shandong Agricultural University, Tai’an 271018, China; niuniu@sdau.edu.cn; 3 College of Agriculture and Forestry Sciences, Linyi University, Linyi 276000, China; xuping3792@lyu.edu.cn; 4 Rizhao Agricultural Technology Service Center, Rizhao 276801, China; wh_gzyx@163.com (H.W.); wangheng0603@126.com (H.W.); 5 School of Life Science, Yantai University, Yantai 264005, China; tyyu@ytu.edu.cn

**Keywords:** peanut, seed coat color, anthocyanin, bHLH, AhWSC1

## Abstract

Seed coat color in peanut (*Arachis hypogaea* L.) is a critical agronomic trait that affects both nutritional quality and market appeal. In this study, we identified two bHLH transcription factor genes, *AhWSC1a* and *AhWSC1b*, homologues of *Arabidopsis TRANSPARENT TESTA 8*, as indispensable gatekeepers of basal flavonoid pigmentation. QTL-seq analysis of a recombinant inbred line population derived from a black-testa parent (S3) and a white-testa parent (S2) revealed that recessive loss-of-function mutations in both *AhWSC1a/1b* abolish proanthocyanidin biosynthesis, resulting in a white testa. Integrated metabolomic and transcriptomic profiling confirmed the absence of proanthocyanidins and a strong repression of late anthocyanin-pathway genes (*DFR*, *LDOX*) in the mutants. Molecular assays further demonstrated that AhWSC1 physically interacts with the R2R3-MYB regulator AhTc1 to form a functional MBW complex that activates *AhDFR* and *AhLDOX* transcription. In this research, we also found that the black testa phenotype may arise from elevated *AhTc1* expression associated with a structural variant (SV); however, in the SV background, the introduction of *ahwsc1a*/*1b* mutant leads to a significant suppression of *AhTc1* expression. Notably, because *AhWSC1* is transcriptionally silent in hairy-root systems, overexpression of *AhTc1* alone failed to induce these late-stage anthocyanin biosynthesis genes, highlighting *AhWSC1* as an indispensable, rate-limiting hub of anthocyanin biosynthesis pathway regulation. Collectively, our findings establish *AhWSC1a* and *AhWSC1b* as master regulators of peanut testa pigmentation, elucidate the molecular basis of classical white testa inheritance, and provide genetic targets for precision-breeding of nutritionally enhanced cultivars.

## 1. Introduction

Seed coat color of peanut (*Arachis hypogaea* L.) is more than a visual trait—it influences seed antioxidant capacity, consumer acceptance, and market price. It is increasingly targeted in breeding programs aimed at developing functional-food cultivars rich in anthocyanins and proanthocyanidins. Flavonoid pigments are strong natural antioxidants [1]. They give peanut seed coats colors that range from pink to purple or black. The accumulation of these flavonoid pigments not only elevates the antioxidant capacity and overall nutritional quality of peanut kernels but also enhances resistance to insect pests, pathogenic microorganisms, and abiotic stresses. However, in peanut, the genetic loci and regulatory networks underlying testa pigmentation remain insufficiently characterized, limiting the development and application of molecular breeding strategies to improve this trait of both nutritional and economic significance.

Significant progress has been made in elucidating the regulatory mechanisms governing anthocyanin biosynthesis in a variety of plant species. Central to this pathway is a conserved transcriptional regulatory module known as the MYB-bHLH-WD40 (MBW) complex [2,3], which orchestrates the expression of key structural genes involved in the late stages of anthocyanin biosynthesis. In *Arabidopsis thaliana*, this complex consists of R2R3-MYB transcription factors (such as *PAP1*/*PAP2*), bHLH TFs (e.g., *TT8*, *GL3*, *EGL3*) [4], and the WD40 repeat protein *TTG1* [5]. Together, these components form a ternary complex that activates genes encoding enzymes such as DFR (dihydroflavonol 4-reductase), ANS (anthocyanidin synthase), and UFGT (UDP-glucose: flavonoid 3-O-glucosyltransferase), which are crucial for anthocyanin accumulation. In rice (*Oryza sativa*), as well as in other economically important crops, substantial progress has been made in deciphering the regulatory mechanisms underlying anthocyanin biosynthesis. Although most cultivated rice varieties produce non-pigmented grains, pigmented types such as black and red rice accumulate high levels of anthocyanins or proanthocyanidins in the pericarp. Key transcriptional regulators include *OsC1* [6], an R2R3-MYB transcription factor homologous to maize *C1*, and bHLH partners such as *OsB1*, *OsB2*, and *OsRb*, which together form a functional MBW complex. Another essential regulator, *Rc*, encodes a bHLH protein controlling proanthocyanidin biosynthesis [7]. Mutations in *Rc* are associated with the white pericarp phenotype in many cultivated rice varieties [8]. These findings demonstrate that MBW-like modules in rice regulate flavonoid pathway genes similarly to those in model systems, though with lineage-specific features.

To date, multiple genes involved in regulating seed coat color have been identified in peanut, shedding light on the molecular basis of testa pigmentation. Among them, a dominant R2R3-MYB transcription factor, *AhTc1*, has been shown to upregulate anthocyanin biosynthetic genes, contributing to the deep purple seed coat phenotype [9]. In red testa varieties, a series of loci collectively referred to as the “red-testa” series includes *AhRt1* on ChrA03 (encoding a bHLH transcription factor), *AhRt2* on ChrB02 (encoding anthocyanidin reductase, ANR), and the more recently identified *AhRt3*, a second ANR homolog [10,11,12]. These loci modulate either the expression levels or enzymatic activity of ANR, thereby redirecting metabolic flux toward or away from cyanidin-derived pigments and resulting in distinct shades of red. In purplish-red peanut lines, the bHLH transcription factor AhPSC1 forms a regulatory complex with AhMYB7 to repress *ANR* expression, further enhancing anthocyanin accumulation [13]. It has been proposed that UFGTs and ANRs compete for the common substrate cyanidin, and that the relative enzymatic activities of these two pathways play a critical role in determining the peanut testa color [14].

Despite recent advances in deciphering the genetic determinants of black and red seed coats, the most prevalent testa color in both wild and cultivated peanuts is pink (also described as light brown), with white variants occurring in rare germplasm [15]. The molecular mechanisms that drive the formation of these common and rare testa colors, however, remain poorly characterized, and the underlying regulatory networks have yet to be systematically unraveled. Therefore, the purpose of this study was to identify the key genetic regulators and to elucidate the regulatory and metabolic mechanisms underlying peanut testa colour variation, with a particular focus on the transition between the predominant pink testa and the rare white testa. In this research, we used a multi-omics approach in combination with a recombinant inbred line (RIL) population derived from a cross between black and white testa to dissect the genetic basis of testa pigmentation. Our analysis identified a pair of paralogous bHLH type transcription factor genes as key regulators responsible for the development of the pink testa phenotype. Notably, recessive homozygous mutations in both of these twin genes result in the complete loss of pigmentation, giving rise to the white testa phenotype. Building on this finding, we further integrated metabolomic and transcriptomic data to systematically dissect the molecular mechanisms underlying testa colour variation in peanut.

## 2. Results

### 2.1. Proanthocyanidin Biosynthesis Is Disrupted in the Seed Coat of Peanuts with White Testa

**Figure 1:**
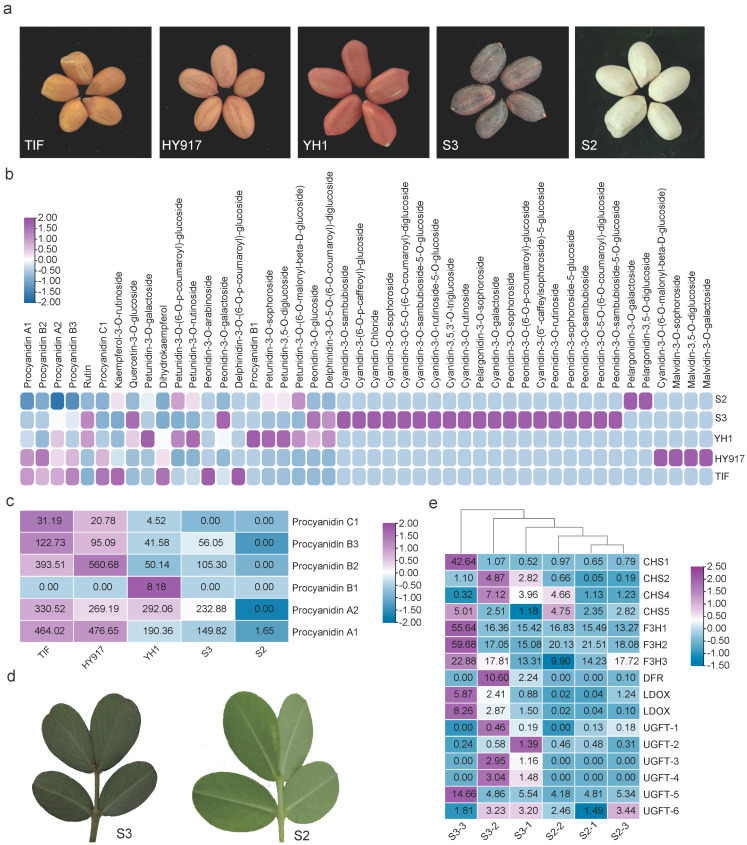
Metabolomic and transcriptomic analysis of peanut varieties with different testa colors. (**a**) Testa color variation among peanut varieties: pink (TIF, HY917); red (YH1); black (S3); white (S2). (**b**) Heatmap of anthocyanin contents in seed coats of different colors (normalized values). (**c**) Heatmap of proanthocyanidin contents in seed coat of different colors; numbers indicate concentrations (ng/mg). (**d**) Leaf color differences between S3 and S2. (**e**) Heatmap of expression levels (FPKM values) for anthocyanin pathway enzyme genes in leaves of S2 and S3.

Seed-coat colors in cultivated peanut include pink, red, black (also termed deep purple), white, and other less common hues. To determine the differences in anthocyanin content among these different colors, we conducted targeted metabolomic analysis on peanuts with pink (HY917, Tifrunner), black (S3), red (YH1), and white (S2) seed coats (Figure 1a). The results revealed marked differences in flavonoid-related metabolites, including anthocyanins and proanthocyanidins, among peanuts with varying seed coat colors (Figure 1b). Compared to pink seed coats, black seed coats exhibited significantly higher levels of various glycosylated forms of anthocyanins than other colored peanuts (Figure 1b, Table S1). Cyanidin-3-(6-O-p-caffeoyl)-glucoside, Cyanidin-3-O-sambubioside, Quercetin-3-O-glucoside and rutin were identified as the predominant form of cyanidin (Figure 1b, Table S1). In contrast, procyanidins were nearly undetectable in white seed coats (Figure 1c, Table S1). Red seed coats showed a significant reduction in procyanidin content compared to pink seed coats, while Quercetin-3-O-glucoside, rutin, and glycosylated forms of petunidin increased significantly (Figure 1b,c, Table S1).

Color difference was also observed in newly emerged young leaves between black (S3) and white (S2) seed coat peanuts (Figure 1d). Petioles and leaves of the white-seeded S2 line accumulated minimal anthocyanins, whereas those of the S3 line displayed markedly deeper pigmentation. Transcriptome analysis of newly emerged leaves from S2 and S3 lines demonstrated that the expression of key metabolic enzymes in the anthocyanin biosynthesis pathway was significantly reduced in the white-seeded S2 peanuts. Enzymes such as dihydroflavonol 4-reductase (DFR) and leucoanthocyanidin dioxygenase (LDOX) were barely detectable in S2 leaves (Figure 1e).

### 2.2. Population Development and Genetic Analysis of Seed Coat Color

**Figure 2:**
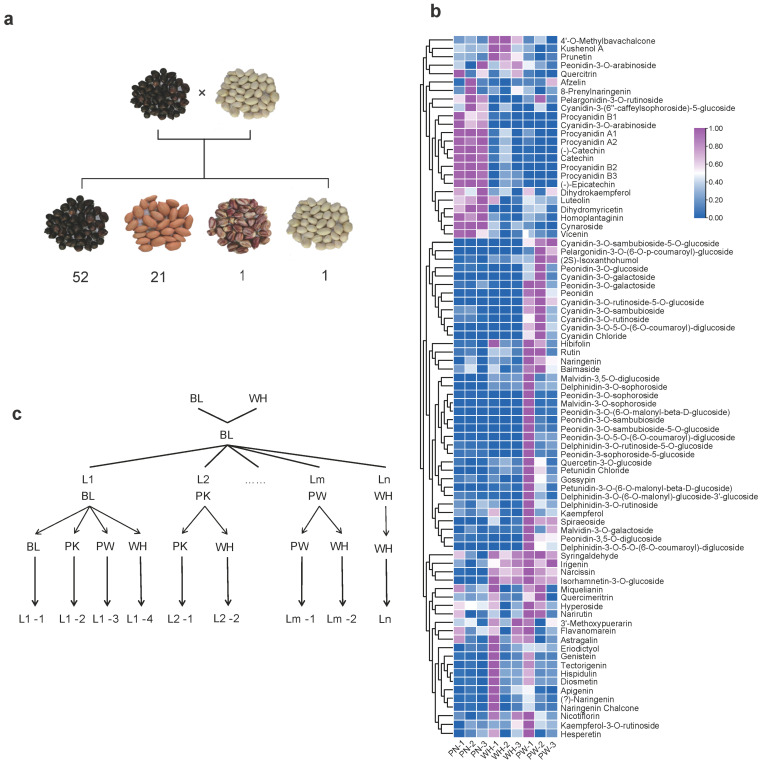
The construction and genetic analysis of the RIL population. (**a**) Segregation of testa colors in F2:3 generation from S3 × S2 cross: black (BL), pink (PN), purple-white (PW), white (WH). (**b**) Analysis of major pigment contents in seed coat of pink (PN), purple-white (PW), white (WH). (**c**) Schematic diagram of RIL population development. The arrows indicate selfing to establish independent lines.

To investigate the mechanism underlying peanut seed coat color formation and anthocyanin metabolism, the white-seeded peanut line S2 and the black-seeded line S3 were used to construct a genetic mapping population. Observation of seed coats in the F2:3 generation revealed that the segregating progeny exhibited black (BL), pink (PN), and white (WH) seed coats, along with a small number of white seeds with purple stripes (PW) (Figure 2a). Metabolomic analysis showed that both PW and WH seeds lacked detectable levels of procyanidins, as well as their precursors catechin and epicatechin (Figure 2b, Table S2).

Analysis of seed coat color in the F3:4 and F4:5 generations demonstrated that the black trait is dominant over pink, pink is dominant over white, and purple-striped white is also dominant over plain white (Figure 2c). Chi-square tests confirmed a 3:1 segregation ratio for black versus pink, and a 15:1 segregation ratio for pink versus white (Table 1). This indicates that, relative to pink peanuts, the black trait is controlled by a single dominant gene, while the white seed coat phenotype is controlled by two recessive genes. Due to the low frequency of white-seeded individuals in the F2 generation, we focused on selecting white-seeded progeny from the F3 and F4 generations within each line for genetic analysis to clone the gene(s) regulating the white seed coat (Figure 2c).

**Figure 3:**
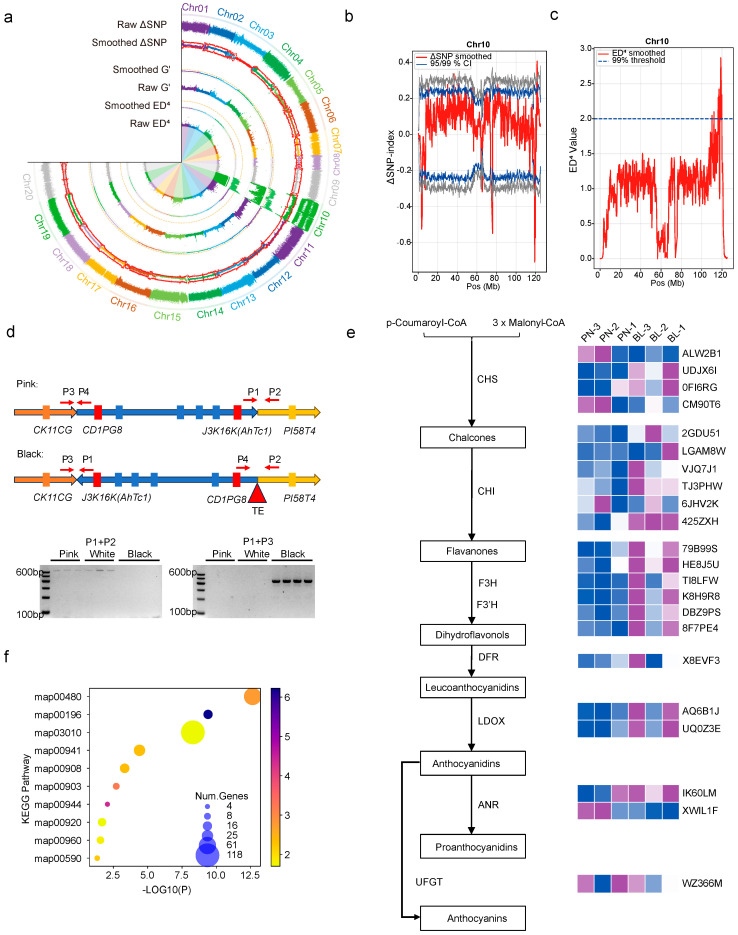
Bulk Segregant Analysis (BSA) mapping of black-testa-related QTL, molecular marker development, and transcriptomic analysis. (**a**) Circos plot of black vs. pink BSA across all chromosomes, generated with multiple algorithms. (**b**) Delta-SNP index plot for Chr10, where the red line shows the smoothed SNP index curve, and the gray and blue lines indicate the 99% and 95% confidence thresholds, respectively. (**c**) QTL mapping on Chr10 based on the ED4 algorithm, with the red line representing the smoothed ED4 curve and the horizontal blue line indicating the 99% confidence threshold. (**d**) Schematic diagram of structural variations associated with the black seed coat in peanut S3 and corresponding PCR validation markers. P1, P2, P3, and P4 are PCR primers flanking the inversion region, showing differences in amplification fragments between black and pink seed coats. (**e**) Heatmap of anthocyanin pathway gene expression in pink (PN), and black (BL) testa. Data were normalized to a 0–1 scale. (**f**) KEGG enrichment analysis of DEGs between black and pink seed coats: circle size indicates the number of DEGs per pathway; the color gradient represents the enrichment fold change; X-axis denotes the *p*-value (−log_10_ scaled). The arrows indicate the direction of the metabolic reactions.

From the F5 generation, twenty phenotypically stable lines were selected from each of the black and pink seed coat categories and pooled separately for BSA based on pooled sequencing. Analysis of the pink and black bulks identified a key quantitative trait locus (QTL) on chromosome Chr10, within the 100–120 Mb interval, that regulates the production of the black seed coat in peanut (Figure 3a–c). Sequence analysis of this interval revealed the presence of *AhTc1* (*Arachis hypogaea Testa color 1*), a gene previously reported as a key regulator of black seed coat formation in peanut. Transcriptomes of black and pink seed coats at the harvest maturity stage (R8) [14] were analyzed (Table S3). Consistent with previous studies, our transcriptome data revealed that *AhTc1* is significantly upregulated in black testa compared to other color classes. However, the underlying mechanism responsible for this elevated expression has remained unclear. In this study, We identified an inversion extending from the upstream region of *AhCD1PG8* to the downstream region of *AhJ3K16K* (*AhTc1*) (Figure 3d and Figure S1). Notably, both *AhCD1PG8* and *AhTc1* are candidate genes for black testa formation in peanuts [9,16]. RNA-seq data indicated elevated *AhTc1* expression specifically in black testae (Table S3). New markers were developed to detect this inversion (Figure 3d). How this inversion directly contributes to the upregulation of *AhTc1* expression need further investigation. In transcriptome analysis, genes involved in flavonoid and anthocyanin metabolism were found to be significantly upregulated in black seed coats (Figure 3e). KEGG pathway enrichment analysis of the upregulated genes showed that the flavonoid biosynthesis pathway (map00941) was among the top ten enriched metabolic pathways (Figure 3f). Heatmap analysis based on the expression levels of major flavonoid biosynthetic enzyme genes further confirmed that these genes were markedly upregulated in developing black seed coats (Figure 3e).

### 2.3. Genetic Mapping and Analysis of the White Seed Coat Loci

**Figure 4:**
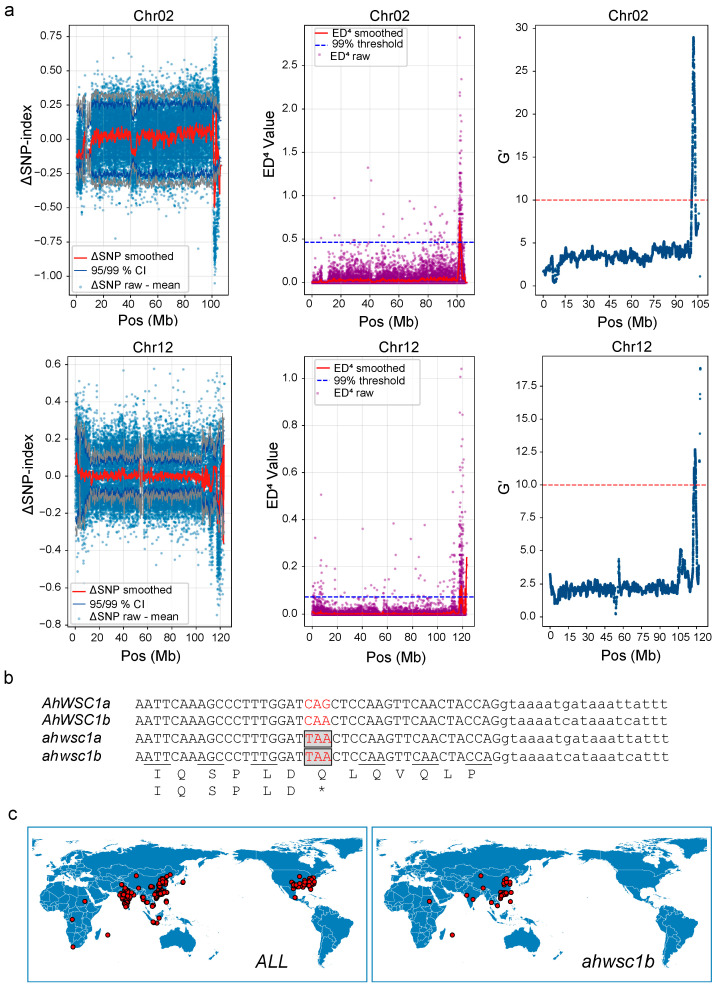
Mapping of the white seed coat gene *AhWSC1*. (**a**) QTLs for white testa on Chr02 and Chr12 identified with ΔSNP-index, ED4, and G′ algorithms: scatter points indicate the raw ΔSNP-index, ED4, or G′ values at each locus; the red curve depicts the smoothed ΔSNP-index or ED4 profile, while the blue curve depicts the smoothed G′ profile. (**b**) Nonsense mutation sites in *AhWSC1a* and *AhWSC1b*; uppercase letters denote exon sequences, lowercase letters denote intron sequences, whereas * represents a stop codon. (**c**) Geographic distribution of the *AhWSC1b* genotype variant across domestic and international cultivars.

The genetic mechanisms underlying white seed coat formation in peanut remain poorly understood. In our genetic mapping population, the white seed coat phenotype was found to be a polygenic recessive trait relative to pink. To investigate the genetic basis of this trait, BSA-seq was performed by comparing pools of white and pink seed coat lines. After applying ΔSNP index, G prime (G′), and Euclidean Distance (ED) algorithms, we detected two highly significant quantitative trait loci (QTLs) on Chr02 and Chr12 (Figure 4a and Figure S2). By comparing SNP profiles and BAM alignment data between the S2 line and the white seed coat bulk, two candidate genomic intervals were identified: Chr02: 97,068,567–99,232,985 and Chr12: 113,267,287–114,173,564. Within these regions, the SNP polymorphism patterns in the white seed coat bulk were found to be completely identical to those of the white parent S2, suggesting a strong genetic correlation. Functional annotation revealed that these QTL intervals are located within collinear regions of the peanut A and B subgenomes (Tables S4 and S5). The candidate intervals encompass 110 and 56 protein-coding genes on Chr02 and Chr12, respectively (Tables S4 and S5). Notably, *Ah.MP3D3D* on Chr02 and *Ah.26781N* on Chr12 were the only genes with nonsense mutations in their coding regions (Figure 4b), and importantly, no other gene pair within the intervals exhibited such homologous mutations across the A and B sub-genomes (Tables S4 and S5). Both *Ah.MP3D3D* and *Ah.26781N* encode homologs of *Arabidopsis thaliana TRANSPARENT TESTA 8* (*AtTT8*), a key regulator of anthocyanin biosynthesis. Accordingly, they were designated *White Seed Coat 1a* (*AhWSC1a* on Chr02) and *White Seed Coat 1b* (*AhWSC1b* Chr12), and were prioritized as strong candidate genes responsible for the white seed coat phenotype. We hypothesize that the white seed coat phenotype may result from concurrent mutations in homologous genes across both subgenomes.

### 2.4. Natural Population Analysis and Transgenic Validation

When aligning to the Tifrunner reference genome, we found that in S2, a potential structural variation may be located near the nonsense mutation site of *AhWSC1a*, and similarly, a structural variation (SV) might also exist near the nonsense mutation of AhWSC1b in S3. However, analysis based on the Shitouqi reference genome revealed that this structural variation is present in both *AhWSC1a* and *AhWSC1b* in S2 (Figure S3). Sequence analysis shows that, in the Tifrunner assembly, *AhWSC1b* carries an intronic insertion that makes this region 67 bp longer than in *AhWSC1a* (Figure S4a). By contrast, in the Shitouqi genome, *AhWSC1a* and *AhWSC1b* differ in this region only at the SNP level. A PCR marker was developed to detect the SV (Figure S4b), and Sanger sequencing revealed that the amplicon derived from the S2 line is 67 bp longer than that from the S3 line and SNP analysis indicated that this marker can simultaneously detect the structural variation in both the A and B subgenomes (Figures S4b and S5). In this study, we refer to the short structural-variant insertion in the *AhWSC1b* allele as SVb, and the analogous insertion in *AhWSC1a* as SVa. Moreover, all white and purple-white testa lines were homozygous for the SVa and SVb allele (Figure S4b). Resequencing data of 390 cultivated peanut accessions was analyzed [17]. It revealed that the SVb in *AhWSC1b* is prevalent, occurring in 191 accessions. Among these accessions, 62 accessions also carry the nonsense mutation, whereas no nonsense mutations were detected in accessions lacking the SVb (Table S4). This distribution pattern supports a hypothesis that the nonsense mutation is a derived allele that arose subsequent to the SV event. In contrast, the SVa linked to the *AhWSC1a* nonsense mutation site was found exclusively in the S2 line and some white peanuts we collected Such as Baiyu, Chi-Bai. SVa and nonsense mutation were absent from all 390 cultivated peanut accessions included in the resequencing panel (Table S4). Moreover, the frequency of the *ahwsc1b* mutation is significantly higher in domestic cultivars than in foreign ones, implying that the genetic base of Chinese breeding lines may be relatively narrow (Figure 4c and Figure S6).

**Figure 5:**
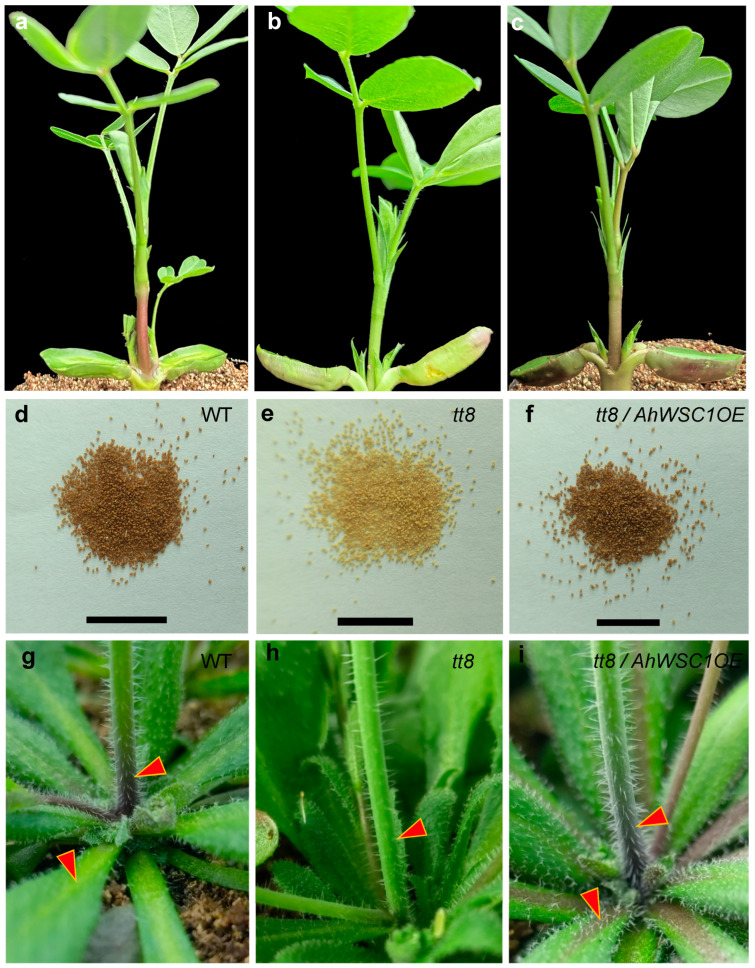
Complementation of *Arabidopsis tt8* mutant by *AhWSC1*. (**a**–**c**) Pigmentation differences in the epicotyls and petioles of newly germinated peanut seedlings: pink (**a**), white (**b**), and black (**c**). (**d**–**i**) Comparison of testa color, epicotyl pigmentation, and rosette-leaf vein pigmentation in wild-type plants (**d**,**g**), the *tt8* mutant (**e**,**h**), and *tt8* plants expressing *AhWSC1a* (**f**,**i**). The black scale bar represents 1 cm. The arrows indicate the main stem and leaf veins.

In *Arabidopsis thaliana*, the *tt8* mutant exhibits reduced pigmentation in the seed coat, primarily due to impaired anthocyanin biosynthesis [4]. In this study, we observed that seedlings from white-seeded peanut lines isolated from the RIL population displayed visibly lighter epicotyls and petioles compared to those from pink and black lines (Figure 5a–c). These phenotypic similarities suggest that the function of *TT8* may be conserved between peanut and *Arabidopsis*. *AhWSC1* was overexpressed in the *Arabidopsis tt8* mutant under the control of the *CaMV 35S* promoter. The results showed that the pale yellow seed coat color of the mutant was restored in the transgenic lines (Figure 5d–f). In addition, purple pigmentation was observed in the petioles of rosette leaves and at the epicotyl base of transgenic *Arabidopsis*, indicating functional complementation of *AhWSC1* in the *attt8* background (Figure 5g–i).

### 2.5. Mechanistic Insights into AhWSC1-Mediated Regulation of Anthocyanin Metabolism

**Figure 6:**
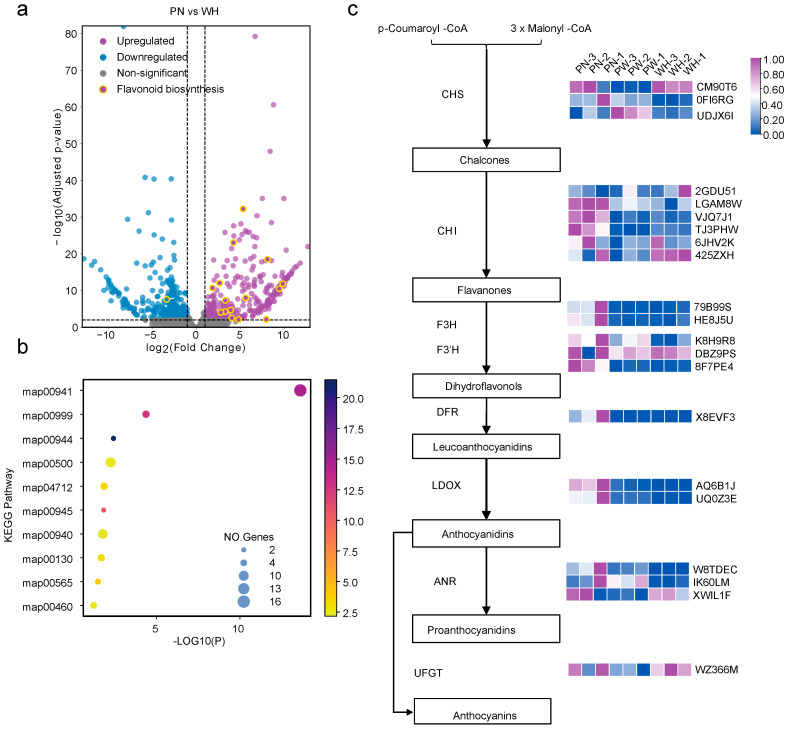
Transcriptomic analysis of anthocyanin pathway genes in RIL testae (pink, white, purple-white). (**a**) Volcano plot of DEGs between pink and white seed coat, with anthocyanin-pathway genes highlighted in yellow. (**b**) Bubble plot of KEGG enrichment for DEGs upregulated in pink seed coats: bubble size reflects the number of DEGs per pathway, color gradient indicates the enrichment fold change, and the X-axis displays −log_10_ (*p*-value). (**c**) Heatmap of anthocyanin pathway gene expression in pink (PN), white (WH), and purple-white (PW) seed coat. Data were normalized to a 0–1 scale. The arrows indicate the direction of the metabolic reactions.

RNA-seq comparison of white, purple-white, and pink seed coat revealed broadly high expression of anthocyanin-biosynthetic genes in the pink seed coat (Figure 6a). KEGG enrichment of the down-regulated set again highlighted flavonoid biosynthesis (map00941) as the most over-represented pathway (Figure 6b). Targeted metabolomics showed that proanthocyanidins were present only at trace levels in the white testa and remained extremely low in the purple-white seed coat (Figure 2b). Integrated clustering of metabolomic and transcriptomic data revealed that white and purple-white seeds share a highly similar expression profile for anthocyanin-pathway genes (Figure 6c).

**Figure 7:**
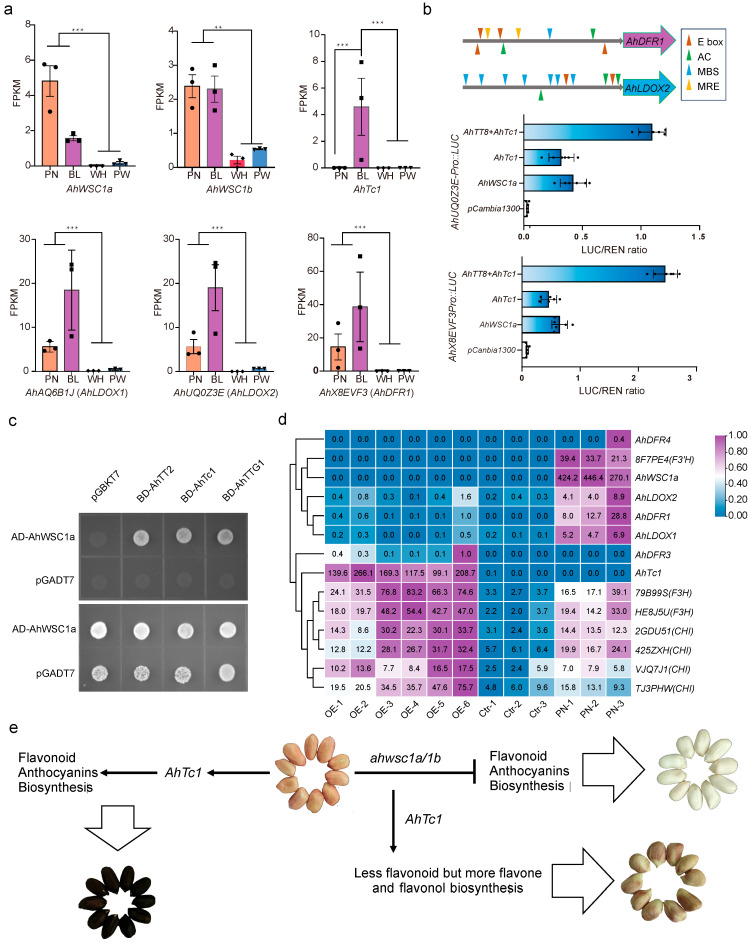
*AhWSC1* is indispensable for testa pigmentation. (**a**) Expression levels (FPKM) of *AhWSC1a*, *AhWSC1b*, *AhTc1*, *AhDFR1*, *AhLDOX1*, and *AhLDOX2* in RILs segregating for seed coat color: black (BL), white (WH), purple-white (PW), and pink (PN). ** indicates a significant difference (*p* < 0.01); *** indicates a highly significant difference (*p* < 0.001). (**b**) Predicted *AhWSC1* binding sites (E-box) and *AhTc1* binding sites (MBS, MRE, AC-box) in promoters of *AhDFR1* and *AhLDOX2*, with dual-luciferase assays showing transcriptional activation. LUC, firefly luciferase; REN, *Renilla* luciferase. (**c**) Yeast two-hybrid assay validating the protein–protein interactions between AhWSC1a and homologues of MBW-complex components. (**d**) Heatmap displaying hierarchically clustered expression levels of DFR, LDOX, and representative early biosynthetic genes. Sample groups are defined as follows: OE-1, OE-2, and similar lines are hairy roots overexpressing *AhTc1*; Ctr-1, Ctr-2, and Ctr-3 lines are non-transformed control roots; PN-1, PN-2, and PN-3 samples correspond to pink testa tissue. The numbers in the heatmap indicate raw FPKM expression values for each gene across different tissues. (**e**) A working model of seed coat colour formation in peanut: Pink represents the basal colour. Homozygous loss-of-function mutations in the twin bHLH TF *AhWSC1a* and *AhWSC1b* block anthocyanin and proanthocyanidin biosynthesis, yielding a white testa. A dominant high-expression mutation in *AhTc1* boosts anthocyanin accumulation, producing a black testa. When this *AhTc1* mutation occurs in the *AhWSC1a/1b* double-mutant background, anthocyanin and proanthocyanidin remain blocked, but flavonols and other bypass-pathway metabolites accumulate, giving rise to an irregular purple-white testa.

In particular, transcripts for dihydroflavonol-4-reductase (*DFR*) and leucoanthocyanidin dioxygenase (*LDOX*) were almost undetectable in both white and purple-white seeds (Figure 7a), consistent with reports that *DFR* and *LDOX* are direct downstream targets of TT8 in other species [2]. A dual-luciferase reporter assay in *Nicotiana benthamiana* leaves confirmed that AhWSC1 strongly trans-activates the promoters of *DFR* and *LDOX* (Figure 7b). Correspondingly, RNA-seq data showed that *DFR* and *LDOX* expression in black testa is substantially higher than in pink (Figure 3f). Co-expression of *AhWSC1* and *AhTc1* in the dual-luciferase system greatly enhanced promoter activity (Figure 7b), and yeast two-hybrid assays revealed a direct physical interaction between the two proteins (Figure 7c).

Although dual-luciferase assays in leaves of *Nicotiana benthamiana* demonstrated that *AhTc1* can activate the expression of *DFR* and *LDOX*, the transcript levels of these genes remain very low in white and purple-white peanut testa (Figure 7a). Molecular marker analysis confirmed the presence of the *AhTc1*-associated structural variant in PW and mutations in both *AhWSC1* homologs. In PW, *AhTc1* expression itself is not significantly elevated and remains suppressed. Thus, in peanut testa, both the expression of *AhTc1* and its activation of downstream targets depend on intact *AhWSC1a* and *AhWSC1b* function. To investigate whether the transcriptional activation of *DFR* and *LDOX* by *AhTc1* depends on *AhWSC1*, we performed transcriptome analysis using a hairy root transformation system. The results showed that *AhWSC1a* and *AhWSC1b* remained undetectable in the hairy roots overexpressing *AhTc1* (Figure 7d). Despite the high expression of *AhTc1* in these roots (FPKM > 100, approximately half the level observed in black testa), *DFR* and *LDOX* transcripts were still nearly undetectable (Figure 7d). In contrast, several upstream genes in the anthocyanin biosynthetic pathway, such as *cinnamate 4-hydroxylase* (*C4H*), *Flavanone 3-hydroxylase* (*F3H*), and *chalcone synthase* (*CHS*), were significantly upregulated (Figure 7c, Table S7).

## 3. Discussion

### 3.1. Structural Variation Associated with High Expression of AhTc1, Resulting in Black Seed Coats

In the recombinant inbred line (RIL) population crossed with black- and white-testa parents, segregation yielded pink testa, indicating that the black phenotype is dominant over pink. Bulked-segregant analysis pinpointed the known dominant locus *AhTc1* as a major contributor to black testa formation [9]. Consistent with previous studies, our transcriptome data show that *AhTc1* is significantly upregulated in black testa compared with other color classes. The mechanism driving this elevated expression had remained elusive, however. In this study, we identified a structural variation near the promoter region of *AhTc1* in black-testa lines (Figure 3d and Figure S1), which may have caused an inversion. Whether this inversion affects *AhTc1* expression still requires further investigation. Consistent with previous studies, our transcriptome data show that *AhTc1* is significantly upregulated in black testa compared with other color classes. The mechanism driving this elevated expression had remained elusive, however. In this study, we identified a structural variation near the promoter region of *AhTc1* in black-testa lines (Figure 3d and Figure S1), which may have caused an inversion. We hypothesize that the inversion could be associated with elevated *AhTc1* expression through more than one regulatory scenario, although direct evidence for the underly ing mechanism is not yet available. One possibility is that, during the inversion, *AhTc1* and its distal regulatory elements (e.g., enhancers) could be brought into a new juxtaposition, such that previously inefficient expression becomes driven by a strong promoter in the new spatial context, leading to elevated transcription [18]. A second possibility is that the inversion alters chromatin folding and 3D genome architecture, repositioning *AhTc1* into an active chromatin domain, thereby increasing its expression [19]. These two mechanisms are not mutually exclusive and may occur simultaneously. These proposed models remain speculative and will require chromatin conformation assays and/or targeted genome editing to be rigorously tested.

### 3.2. AhWSC1 Defines the Foundational Pigmentation Program of Peanut Testa

Peanut displays a striking array of testa colors: black, red, wine (maroon), purple, pink and white. The genetic basis of peanut seed-coat coloration has perplexed researchers for more than 100 years [15]. Classical segregation studies established that testa pigmentation is a complex, polygenic trait, and that pink, occasionally manifesting as a light tan, represents the basal hue [15]. Red testa can originate via two distinct genetic routes: a dominant allele *R1* or recessive mutations at two loci, *r2* and *r3* [20,21]. Purple coloration is conferred by the dominant gene P [22]. Recent advances suggest two distinct genetic routes to red testa pigmentation. In recessive red testa lines, loss-of-function mutations in the anthocyanidin reductase (ANR) genes *AhRt2* and *AhRt3* appear to drive the accumulation of red pigments [11,12]. In contrast, dominant red testa is linked to *AhPSC1*, which encodes a bHLH transcription factor carrying an EAR motif [13]. Acting as a transcriptional repressor, *AhPSC1* likely attenuates ANR expression, thereby limiting proanthocyanidin biosynthesis and shifting the metabolic flux toward red pigmentation.

We note that SV detection at the *AhWSC1a*/*AhWSC1b* locus is dependent on the reference assembly because the Tifrunner and Shitouqi genomes differ in local haplotype structure in this region. In the Tifrunner reference, the insertion is already present in AhWSC1b; therefore, when mapping to Tifrunner, this feature is absorbed by the reference and only the difference in *AhWSC1a* is called as an SV in white-testa materials. In contrast, when using Shitouqi as the reference, the corresponding segment does not contain the same insertion configuration, enabling SV signals to be detected in both *AhWSC1a* and *AhWSC1b*. Importantly, our origin analysis indicates that the *AhWSC1b* SV predates the emergence of the nonsense mutation, and we currently have no evidence that the SV is mechanistically required for generating the nonsense allele. Thus, we interpret the SV primarily as a linked structural feature/marker within the local haplotype background, whereas the nonsense mutations remain the most parsimonious causal lesions for the white testa phenotype.

In addition, Sanger sequencing of S2 showed that *AhWSC1a* and *AhWSC1b* carry the same insertion-type SV and the same nonsense mutation in S2. However, resequencing of the broader natural population indicated that the corresponding SV insertion is not observed at *AhWSC1a* in other accessions, suggesting that the S2 *AhWSC1a* configuration is unusual and likely derived. Notably, Sanger sequencing further revealed that homeolog-specific SNPs flanking the SV/nonsense sites are still present between the two subgenomes, arguing against a large-scale homogenization event. Together, these observations are consistent with the possibility that, in the white-testa line S2, the *AhWSC1a* nonsense mutation and the associated SV may have arisen via a small tract inter-subgenomic recombination/gene-conversion-like event between the two homeologs [23], although the exact mechanism will require additional haplotype-resolved long-read evidence to be confirmed.

Pink (or light tan) is conditioned by two pairs of completely dominant duplicate genes, F1/F2 and D1/D2 [24]. When a dominant allele is present at each pair, the seed coat expresses its basic color [24], Homozygosity for recessive alleles at one or both duplicate pairs, *f1f1 f2f2 D1D1 D2D2*, *F1F1 F2F2 d1d1 d2d2* or *f1f1 f2f2 d1d1 d2d2*, produces white seed coat [24,25,26]. Yet the molecular basis of the basal pigmentation pathway has remained unresolved. In this research, bulked-segregant analysis reveals that concurrent loss-of-function mutations in two homeologous bHLH genes, *AhWSC1a* and *AhWSC1b*, eliminate anthocyanin accumulation, yielding a white testa. This dual-gene model dovetails with earlier genetic predictions (duplicate recessive control) and positions *AhWSC1* as a pivotal regulator of the baseline testa colour in peanut.

### 3.3. AhWSC1 Acts as a Key Regulatory Controlling Proanthocyanidins Biosynthesis

Targeted metabolomic profiling of anthocyanin-related compounds across seed-coat color classes showed a clear pattern: pink testa contained markedly higher levels of proanthocyanidins than any other colour, whereas both red and black testa exhibited reduced proanthocyanidin content (Figure 1). Because anthocyanidins serve as common precursors for the biosynthesis of anthocyanins and proanthocyanidins. Anthocyanidin reductase (ANR) catalyzes the conversion of anthocyanidins into proanthocyanidins, the decrease observed in red testa is plausibly attributable to lower ANR expression. In black testa, proanthocyanidin depletion is more likely driven by metabolic diversion of anthocyanidins toward glycosylated anthocyanins, thereby reducing the substrate pool for proanthocyanidin synthesis. UDP-glucose:flavonoid 3-O-glucosyltransferase (UFGT) plays a key role in anthocyanin biosynthesis, as it catalyzes the glycosylation of unstable anthocyanidins, thereby converting them into stable anthocyanins [27]. By contrast, proanthocyanidin production is almost completely abrogated in white testa (Figure 1b and Figure 2b). Given that blocking ANR alone produces a red testa, the metabolic lesion responsible for the white phenotype must reside further upstream in the pathway. Transcriptome data support this notion: most genes in the anthocyanin pathway are down-regulated, and transcripts of *DFR* and *LDOX* are virtually undetectable in white testa. Dual-luciferase assays confirmed that the bHLH factor *AhWSC1* can activate the promoters of both *DFR* and *LDOX* in tabaco, underscoring its pivotal role as an upstream regulator of anthocyanin biosynthesis.

### 3.4. The Function of AhTc1 Depends on AhWSC1

In addition to the commonly observed white, pink, and black seed coat colors, a small number of recombinant inbred lines (RILs) exhibited a purple-white seed coat phenotype. Metabolomic analysis revealed that the biosynthesis of proanthocyanidins remained blocked in these purple-white peanuts. Genotypic analysis showed that these lines carried double mutations in *AhWSC1a* and *AhWSC1b*, along with the structural variant of *AhTc1*. However, the expression level of *AhTc1* in the purple-white seed coat was only slightly higher than that in white and pink seed coats (Figure 7a). We speculate that *AhTc1* may be highly expressed in only a limited subset of integument cell lineages, leading to the upregulation of some upstream anthocyanin biosynthetic genes in a portion of the cells. The purple-white coloration might result from the blockage of anthocyanin and proanthocyanidin biosynthesis, which causes metabolic flux to be redirected toward other flavonoid-derived pigments, such as flavonols and flavones.

In dicotyledonous plants like *Arabidopsis*, anthocyanin biosynthetic genes are divided into early biosynthetic genes (EBGs) and late biosynthetic genes (LBGs) [28]. EBGs, such as *CHS*, *CHI*, *F3H*, *F3′H*, and *FLS*, are activated by *R2R3*-*MYB* transcription factors and contribute to the production of flavonols and other flavonoids [29]. LBGs, including *DFR*, *ANS*/*LDOX*, and *UFGT*, act downstream and are regulated by the MBW complex to complete anthocyanin synthesis [29]. A similar regulatory mechanism appears to operate in peanut. In *Arabidopsis*, AtTT8 interacts with AtPAP1, AtPAP2, AtMYB113, and TT2 [30]. In this study, yeast two-hybrid assays also demonstrated an interaction between AhTc1 and AhWSC1a. We further found that in white peanut seed coats, the transcription of DFR and LDOX was blocked compared to pink seed coats, while the expression of early biosynthetic genes (EBGs) such as *CHS*, *CHI*, *F3H*, *F3′H*, and *FLS* remained largely unaffected. In *AhTc1*-overexpressing hairy roots, EBGs were significantly upregulated, but *DFR* and *LDOX* transcription was not induced (Figure 7d).

## 4. Material and Methods

### 4.1. Population Development and Phenotypic Assessment

The black-testa cultivar, S3, was crossed with a white-testa cultivar, S2, in 2018 at the experimental station of Shandong Peanut Research Institute, Laixi, Shandong, China to generate the S2 × S3 RIL population (SS population). F_1_ seeds were sown in the same field the following season, and self-pollinated progeny were advanced to the F_5_ generation. All generations (F_1_–F_5_) were grown under identical agronomic management: 70 cm row spacing, 20 cm plant spacing. Because peanut testa color is maternally determined, the testa of F_3_ seeds reflects the genotype of the F_2_ plant. Consequently, seed harvested from each F_2_ to F_5_ individual was visually inspected under daylight and classified into four discrete colour classes—black, white, pink, or purple-white. Segregation ratios were tested by χ^2^ goodness-of-fit, with *p* < 0.05 considered significant.

### 4.2. Genomic Resequencing and BSA

Genomic DNA was isolated from young leaflets of F_5_ plants whose testa color was stable across two seasons. Three bulks were assembled: black, white, and pink. For each bulk, equimolar DNA from each line was pooled to 5 µg total for next generation resequencing. Raw reads were quality-trimmed with fastp v0.23.2, then aligned to the Tifrunner reference genome (v2.0) using Hisat2 with parameter “--no-spliced-alignment”. After duplicate removal and base-quality recalibration, SNPs and indels were called jointly across bulks with GATK HaplotypeCaller in GVCF mode [26,31]. SNP positions with total depth < 10× in any bulk were discarded. Three complementary metrics were applied to the resequencing data: (i) the ΔSNP-index, defined as the difference between the SNP-index of the high-trait bulk and that of the low (or parental mean) bulk [32,33]; (ii) G′ (Gprime), the smoothed G statistic obtained by tricube-kernel weighting [33,34]; and (iii) the fourth-power Euclidean distance (ED^4^), which was modified to increase the signal to noise ratio by raising normal ED to the fourth power [35,36]. For each statistic, values were smoothed in a 1 Mb sliding window with 100 kb steps, and empirical 95% significance thresholds were generated from 100,000 Monte-Carlo permutations of bulk labels [37]. All calculations were implemented in Python v3.10 scripts we developed, which are available on Github (https://github.com/sdpeanut/BSAtool, accessed on 18 June 2025).

### 4.3. Geographic Distribution Analysis of Varieties

We reanalyzed 390 resequencing datasets [17], aligning reads to the Tifrunner reference genome with HISAT2 and inspecting *AhWSC1* mutation types one by one using the IGV v2.17.4 tools [38,39]. Using the associated geographic coordinates, we developed a Python tool, GeoMapDraw v1.0 (https://github.com/sdpeanut/GeoMapDraw, accessed on 18 June 2025), to visualize the geographic distribution of the different mutation types.

### 4.4. De Novo Assembly of S3 and PCR Marker Development

Cleaned reads of S3 were re-assembled de novo using MEGAHIT v1.2.9 on a 96-thread Ubuntu 22.04 server (512 GB RAM) with multi-k-mer mode (--k-min 31 --k-max 141 --k-step 10), 85% memory usage, and a minimum contig length of 1 kb [40]. Known sequences flanking the *AhTc1*-associated SV in S3 were used as BLAST+ v2.12.0 queries to identify the corresponding contigs in the assembly, and primers were designed on the basis of the alignment results.

### 4.5. Targeted Metabolomic Profiling of Anthocyanins

Mature dry seeds were deshelled, and testa were peeled, flash-frozen in liquid nitrogen, and lyophilised for 48 h. Powdered samples (50 mg) were extracted in 1 mL 70% methanol containing 0.1% formic acid, vortex-mixed 30 min at 4 °C, and centrifuged (12,000× *g*, 10 min). Supernatants were filtered (0.22 µm PTFE) before analysis. Three biological replicates per color class were prepared. Chromatographic separation utilized an ACQUITY UPLC HSS T3 column (2.1 × 100 mm, 1.8 µm, Waters, Milford, MA, USA) on a Waters UPLC-I Class system. Mobile phase A: 0.1% formic acid in water; phase B: acetonitrile. Detection used a Sciex QTRAP 6500+ LC–MS/MS system (SCIEX, Framingham, MA, USA), operated as triple-quadrupole in MRM mode, positive ionization, source temperature 550 °C, ion spray voltage 5500 V. A 52-compound anthocyanin standard library were purchased from Sigma-Aldrich (St. Louis, MO, USA) enabled absolute quantification; data were processed with MultiQuant v3.0.3 (https://sciex.com/products/software/multiquant-software, accessed on 20 July 2025).

### 4.6. Transcriptome Sequencing and Analysis

Total RNA was extracted from the first fully expanded leaves of S2 and S3 and from testa at the R9 stage [14] resenting four classes (white, black, pink, purple-white) in the RIL population. RNA libraries were sequenced on Illumina NovaSeq 6000 platform by Biomarker Technologies (Beijing, China). FastQC v0.12.1 was used for quality control. Reads were trimmed with fastp and aligned to the Tifrunner reference transcriptome using HISAT2 v2.2.1 [41,42]. Gene counts were obtained with featureCounts v2.0.3 and normalized to TPM. Differential expression was determined with DESeq2 (https://github.com/thelovelab/DESeq2, accessed on 19 April 2025). KEGG pathway mapping used KOBAS v2.0 [43] and KEGG enrichment employed ClusterProfiler, v2.13 [44].

### 4.7. Arabidopsis Genetic Complementation and Hairy Root Transformation of Peanut

*Arabidopsis thaliana* ecotype Columbia-0 (Col-0) and the *tt8-6* mutant (GABI_241D05) were grown in 7 cm pots filled with peat/vermiculite (3:1) under a 16 h light/8 h dark photoperiod, 22 °C, 60% relative humidity, and ~150 µmol m^−2^ s^−1^ light strength. The full-length *AhWSC1a* coding sequence was amplified from S3 cDNA and cloned into *pCAMBIA1300* under the CaMV 35S promoter, yielding *p35S:AhWSC1a*. The construct was introduced into the Agrobacterium tumefaciens strain GV3101 and transformed into *tt8-3* plants using the floral-dip method [45]. T_0_ seeds were surface-sterilized and selected on MS medium containing 25 mg L^−1^ hygromycin. Integration of the transgene was confirmed by PCR. T_1_ plants were examined for seed-coat pigmentation. Complementation was inferred when the pale yellow testa of *tt8-6* converted to brown. The *AhTc1* coding sequence (CDS) was cloned into the pCAMBIA1300 vector under the control of the CaMV 35S promoter to generate an *AhTc1* overexpression construct (OE). An empty vector lacking *AhTc1* was used as the control (Ctr). Peanut leaves were transformed using Agrobacterium rhizogenes. The resulting hairy roots were harvested for RNA-seq, and transcriptome data from pink-peanut seed coats at the R9 stage (PN) were also included for integrated analysis to identify downstream genes. *Rhizobium rhizogenes* strain K599 was used in transformation of peanut leaf to generate hairy root [46,47].

### 4.8. Y2H and Transcriptional Activation Analysis

The coding sequences (CDSs) of *AhWSC1a*, *AhTT2*, *AhTTG1*, and *AhTc1* were individually cloned into *pGADT7* and *pGBKT7* and co-transformed into *Saccharomyces cerevisiae* strain Y2HGold [48]. To suppress auto-activation, 2 mM 3-AT was added to the selective medium. To assess whether *AhWSC1* activates the promoters of *AhDFR* and *AhLDOX1*, a dual-luciferase reporter assay was conducted in *Nicotiana benthamiana* leaves. The *AhWSC1a* and *AhTc1* coding sequence was cloned into the *pCambia1300* effector vector, and ~2 kb promoter regions of *AhDFR1* and *AhLDOX1* were inserted upstream of the firefly luciferase (*LUC*) gene in the *pGreenII 0800-LUC* reporter vector [49]. The *Renilla* luciferase (*REN*) gene under the CaMV 35S promoter served as an internal control. *Agrobacterium tumefaciens* GV3101 strains carrying the effector and reporter constructs were co-infiltrated into *N. benthamiana* leaves, and proteins were extracted for luciferase activities three days later [50,51]. Dual Luciferase Reporter Assay Kit (Cat# DL101-01, Vazyme, Nanjing, China) were used to perform the assay. The relative promoter activity was expressed as the *LUC*/*REN* ratio, with 6 biological replicates for each assay at least.

## 5. Conclusions

In this study, we demonstrated that two bHLH transcription factor genes *AhWSC1a* and *AhWSC1b* are indispensable regulators of peanut testa pigmentation. Recessive loss-of-function mutations in both loci abolish proanthocyanidin biosynthesis and repress late anthocyanin-pathway genes, resulting in the classical white testa phenotype. In addition, structural variation tightly linked to AhTc1 contributes to the black testa phenotype, yet its effect depends on the presence of functional *AhWSC1*. We further showed that AhWSC1 physically interacts with the MYB regulator AhTc1 to form a functional MBW complex that activates key downstream genes, establishing AhWSC1a/1b as the central transcriptional hub of the anthocyanin pathway. Collectively, these findings elucidate the molecular basis of white testa inheritance in peanut and highlight *AhWSC1a*/*1b* as critical genetic targets for precision-breeding of nutritionally improved peanut varieties.

## Figures and Tables

**Table 1:** Chi-square test for the number of genes controlling different seed coat colors.

Phenotype	Number of Gene	Ratio	Expect	χ^2^	χ^2^_0.05_ (1)	*p*-Value
BL:NB	1	23:52	1:3	1.28	3.84	0.2571
BL:NB	2	23:52	1:15	76.290	3.84	2.5 × 10^−18^
WH:NW	1	1:74	1:3	22.40	3.84	2.22 × 10^−6^
WH:NW	2	1:74	1:15	3.09	3.84	0.0786
WH:NW	3	1:74	1:63	0.0256	3.84	0.8728
WH:NW	4	1:74	1:255	1.7127	3.84	0.1907
WH:PN	1	1:21	1:3	4.909	3.84	0.026
WH:PN	2	1:21	1:15	0.109	3.84	0.741
WH:PN	3	1:21	1:63	1.312	3.84	0.252
WH:PN	4	1:21	1:255	9.761	3.84	0.0018

BL, Black; PN, Pink; NB, Non-black; NW, Non-white; WH, White.

## Data Availability

All the original resequencing data can be available at the web site of the National Genomics Data Center of China National Center for Bioinformation (CNCB) (https://ngdc.cncb.ac.cn/, accessed on 15 January 2025) with the project ID PRJCA021788.

## Supplementary Materials

The following supporting information can be downloaded at https://www.mdpi.com/article/10.3390/plants15020304/s1, Figure S1: Structural variation associated with seed-coat color in black peanut; Figure S2: Circos plot displaying genome-wide QTL scans from the ED4, ΔSNP-index, and G′ algorithms; Figure S3: Reference-genome choice affects the detection of structural variation adjacent to the point-mutation site of *AhWSC1*; Figure S4: Comparative alignment of subgenome sequences flanking the point-mutation sites in *AhWSC1a* and *AhWSC1b* and development of molecular markers; Figure S5: Sanger sequencing chromatogram of the marker amplicon in single white peanut individual, confirming that the fragment derives from both the A and B subgenomes; Figure S6: Number of domestic and international germplasm accessions carrying either the SVb structural variant or the ahwsc1b loss-of-function genotype; Figure S7: Number of domestic and international germplasm accessions carrying either the SVb structural variant or the *ahwsc1b* loss-of-function genotype; Table S1: Content of anthocyanins (ng/mg) in varieties with different seed coat colors; Table S2: Metabolomic profiling of flavanoids and anthocyanins in seed coats from color-segregated Individuals of RIL opulations; Table S3: Transcriptomic analysis of seed coats at R8 stage: black (BL), pink (PN), white (WH), and purple-white (PW); Table S4: Protein-coding genes within the Chr02 QTL interval; Table S5: Protein-coding genes within the Chr02 QTL interval; Table S6: Analysis of different mutation types in natural populations; Table S7: RNA sequencing analysis of flavonoid pathway genes across multiple tissue types; Table S8: Marker primer list.

## Abbreviations

The following abbreviations are used in this manuscript:

DFR	Dihydroflavonol 4-reductase
LDOX	Leucoanthocyanidin dioxygenase
ANS	Anthocyanidin synthase
LUC	Firefly luciferase
REN	Renilla luciferase
ED	Euclidean distance
ED4/ED^4^	Euclidean distance to the 4th power
bHLH	basic helix–loop–helix
BSA	Bulked Segregant Analysis
QTL	Quantitative Trait Locus
CDS	Coding Sequence
