# Supplementary material for: Two bHLH Transcription Factor Genes AhWSC1a and AhWSC1b Act as Gatekeepers of Testa Pigmentation, Preventing White Seed Coats in Peanuts
Source: Plants (Basel). 2026 Jan 20;15(2):304. doi: 10.3390/plants15020304 (PMC12845083; doi:10.3390/plants15020304)
Supplement: Supplementary file 1 [file plants-15-00304-s001.zip › Supplematary Figures MDPI.pdf]

(a)

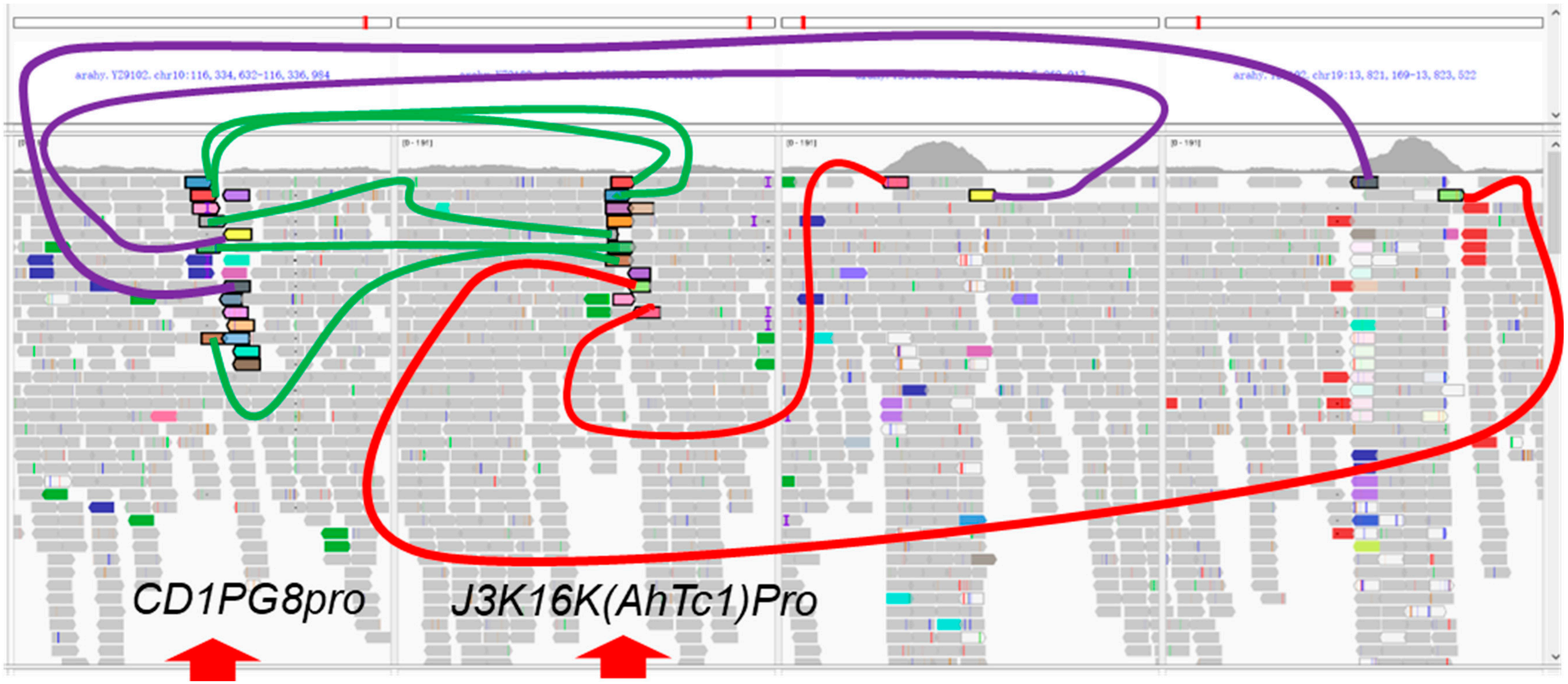

Figure S1. Structural variation associated with seed-coat colour in black peanut.
Reads highlighted in colour represent mate-pair reads whose partners align to genomic regions different from the current read position; the coloured arcs trace the pairing relationships.

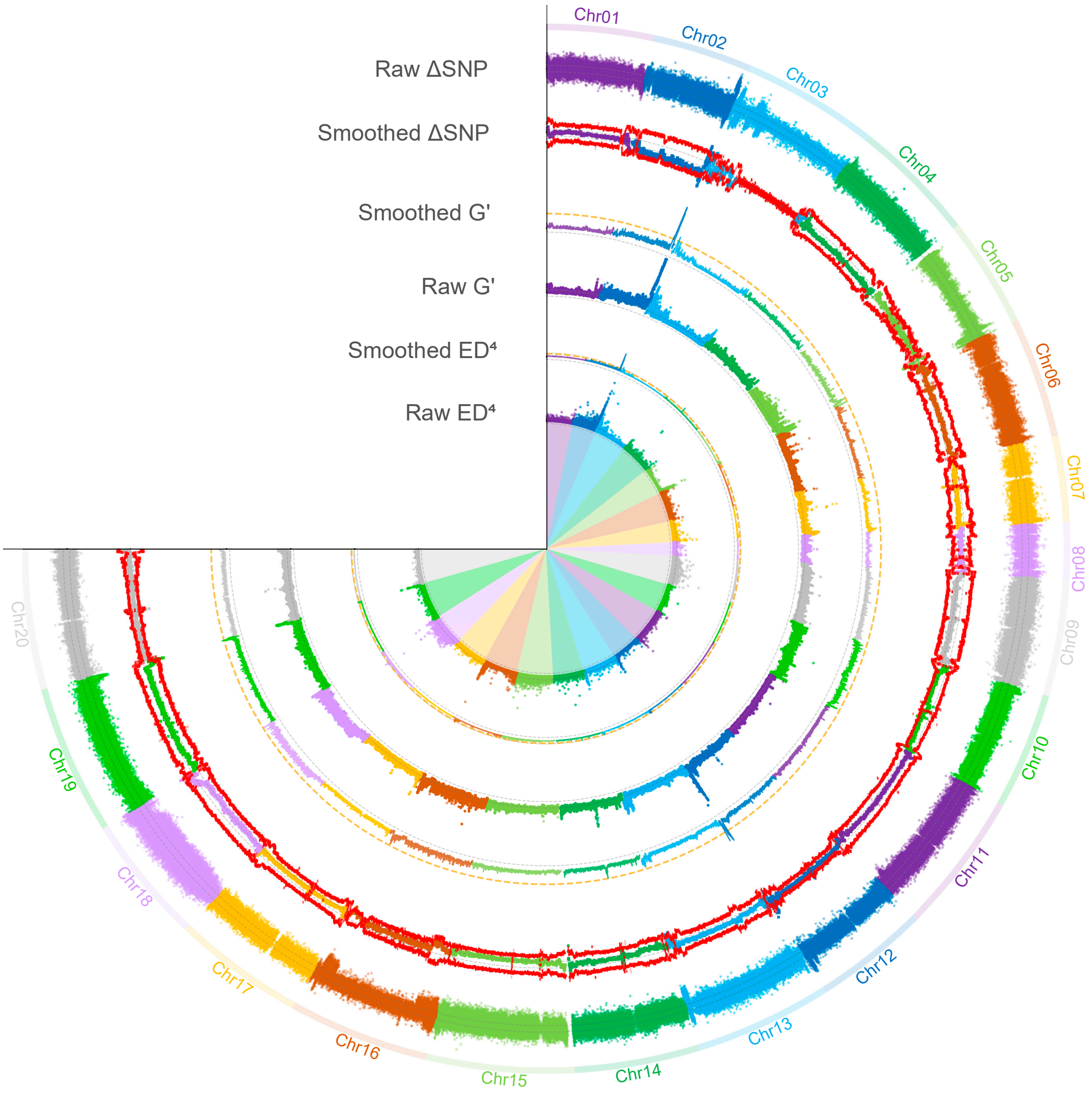

Figure S2. Circos plot displaying genome-wide QTL scans from the ED4, ΔSNP-index, and G′ algorithms; separate tracks show the raw data and their corresponding smoothed profiles across all chromosomes.

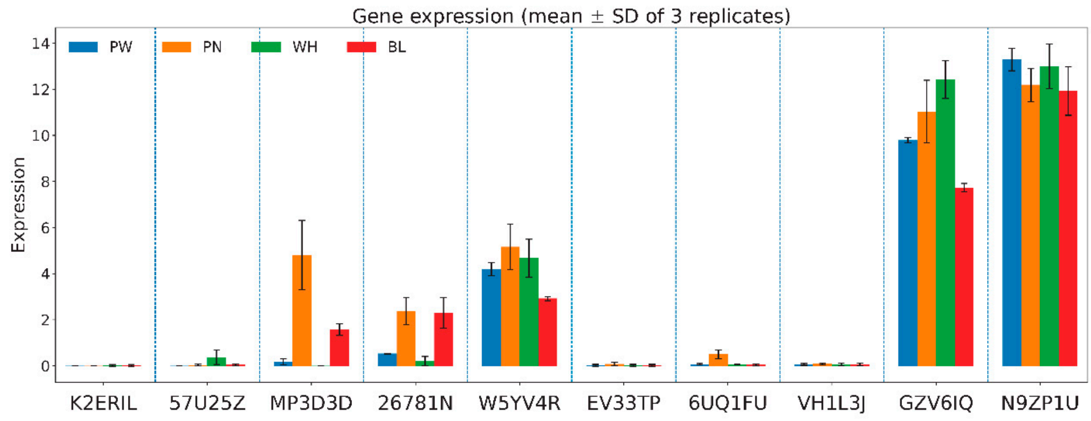

Figure S3. Expression pattern of genes containing SNPs in the CDS region

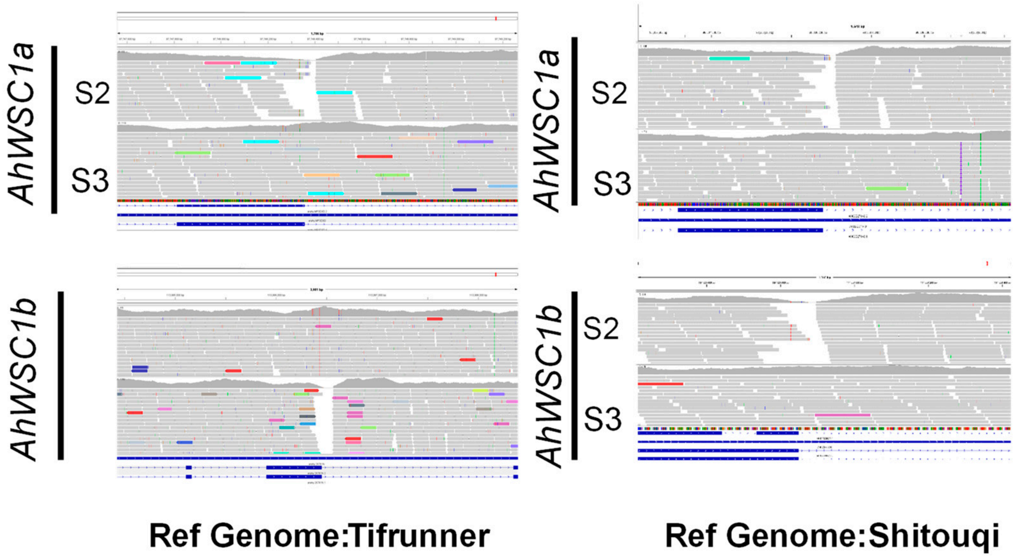

(a) (b)

Figure S4. Reference-genome choice affects the detection of structural variation adjacent to the point-mutation site of *AhWSC1*.

Alignment to the Tifrunner reference genome revealed a structural variant (SV) in *AhWSC1a* of S2 and in *AhWSC1b* of S3. When the Shitouqi reference genome was used, SVs were detected in both *AhWSC1a* and *AhWSC1b* of S2, whereas no SV was observed in S3.

A

```
AhWCS1a  AATTACAGAACCAAGTGAGTTGATGCAATTAGAAATGTCTGAAGATATTAGAGTTGGATC
AhWCS1b  AATTACAGAACCAAGTGAGTTGATGCAATTAGAAATGTCTGAAGATATTAGAGTTGGATC

AhWCS1a  ACCTAATGACGGATCAAATAATCTTGATTCCAATTTTCATTTACTAGCCGTTACCGAAGC
AhWCS1b  ACCTAATGATGGATCAAATAATCTTGATTCCAATTTTCATTTACTAGCCGTTACCGAAGC

AhWCS1a  AGATAATCAGTCGGGTCAAGTCGACTCTTATCGAGTTGACCCGACCCAAAGATGGGATCC
AhWCS1b  AGATAATCATTCGGGTCAAGTTGACTCTTATCGAGTTGACCCGACCCAAAGATGGGATCC

AhWCS1a  AATTCAAAGCCCTTTGGATCAGCTCCAAGTTCAACTACCAGGTAAAATGATAAATTATTT
AhWCS1b  AATTCAAAGCCCTTTGGATCAACTCCAAGTTCAACTACCAGGTAAAATCATAAATCATTT

AhWCS1a  ATTTAA---------------------TTTACTTGTTATAAAATTATGCA----------
AhWCS1b  ATTTAGTTTTTATTAAATATTTTTTATTTTAGTTGTTATAAAATTATTAAAAATTAAAAA

AhWCS1a  -----------------------------------AAAATGTTGATACGTCAAGAAGCAT
AhWCS1b  TTAATTATTTAATTTTCACAGTAAATTTGTTTTGCAAAATGTTGATACGTCAAGAAGCAT

AhWCS1a  GCAAATACCATTTTAATATTTAATTAGCATCTATCAAATAATTTGTTTGTTTGCCAAAAT
AhWCS1b  GCAAATACCATTTTAATATTTAATTAGCATTTATCAAATAATTTGTTTGTTTGCTAAAAT

AhWCS1a  ATTCATGTATCAAGATATATGCATTATTTAAATATTTAACCGATCACAACAATATTTAAC
AhWCS1b  ATTCATGTATCAAGATATATGCATTATTTAAATATTTAACCGATCACAACAATATTTAAC

AhWCS1a  CATGAGAACTAAAATAAATAATTTTTAGAGCAAATACATTTTTTGATTTATGATTATTTG
AhWCS1b  CATGAGAACTAAAATAAATAATTTTTAGAGCAAATACATTTTTTGATTTATGATTATTTG
```

B

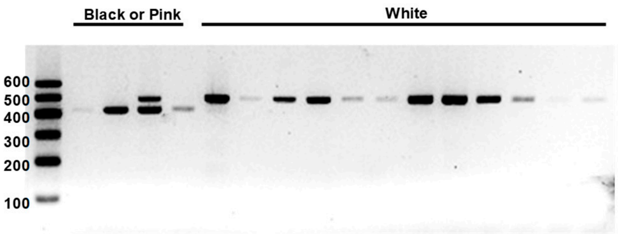

Figure S5. Comparative alignment of subgenome sequences flanking the point-mutation sites in *AhWSC1a* and *AhWSC1b* and development of molecular markers.

(a) Alignment of the Tifrunner subgenome sequences. Green highlights indicate PCR-marker primer binding sites, yellow marks the point-mutation sites, and red denotes SNP differences between the two subgenomes.

(b) PCR amplification of individual segregants with the developed marker. White-testa plants yield only the larger amplicon, whereas pink- and black-testa plants display at least one smaller amplicon.

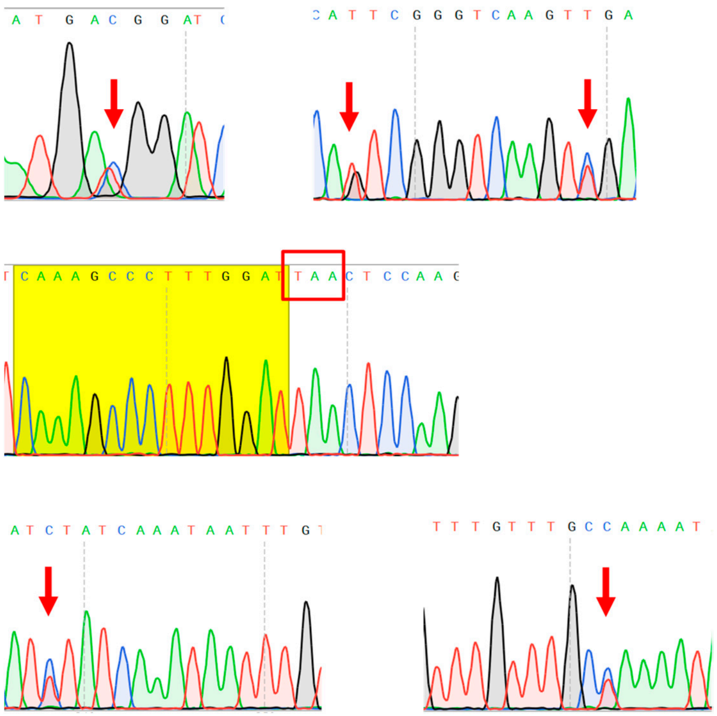

Figure S6. Sanger sequencing chromatogram of the marker amplicon in single white peanut individual, confirming that the fragment derives from both the A and B subgenomes.

Arrows indicate the SNP sites and the point-mutation site highlighted in Figure S4, all of which appear as overlapping (double) peaks.

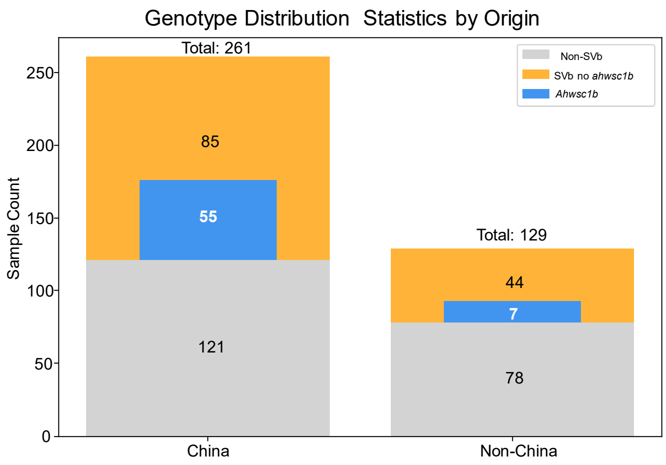

Figure S7. Number of domestic and international germplasm accessions carrying either the SVb structural variant or the ahwsc1b loss-of-function genotype.
